# Role of Diet–Microbiome Interaction in Gastrointestinal Disorders and Strategies to Modulate Them with Microbiome-Targeted Therapies

**DOI:** 10.1146/annurev-nutr-061121-094908

**Published:** 2023-05-28

**Authors:** Ajita Jadhav, Aditya Bajaj, Yang Xiao, Manasvini Markandey, Vineet Ahuja, Purna C. Kashyap

**Affiliations:** 1 Enteric Neuroscience Program, Division of Gastroenterology and Hepatology, Department of Medicine, Mayo Clinic, Rochester, Minnesota, USA;; 2 Department of Gastroenterology and Human Nutrition, All India Institute of Medical Sciences, New Delhi, India;

**Keywords:** gut–brain axis, microbiota, inflammation, metabolomics, fermentation

## Abstract

Diet is an important determinant of health and consequently is often implicated in the development of disease, particularly gastrointestinal (GI) diseases, given the high prevalence of meal-related symptoms. The mechanisms underlying diet-driven pathophysiology are not well understood, but recent studies suggest that gut microbiota may mediate the effect of diet on GI physiology. In this review, we focus primarily on two distinct GI diseases where the role of diet has been best studied: irritable bowel syndrome and inflammatory bowel disease. We discuss how the concurrent and sequential utilization of dietary nutrients by the host and gut microbiota determines the eventual bioactive metabolite profiles in the gut and the biological effect of these metabolites on GI physiology. We highlight several concepts that can be gleaned from these findings, such as how distinct effects of an individual metabolite can influence diverse GI diseases, the effect of similar dietary interventions on multiple disease states, and the need for extensive phenotyping and data collection to help make personalized diet recommendations.

## 1. INTRODUCTION

Diet is an important determinant of an individual’s state of health. The role of diet as a driver of gastrointestinal (GI) symptoms is well understood in conditions such as malabsorption (e.g., lactose intolerance) and specific immune-mediated disorders (e.g., celiac disease, eosinophilic esophagitis, and food allergy). However, people with these disorders constitute a small part of the population who present with food-related symptoms. In a recent survey, nearly 52% of respondents reported meal-related abdominal pain, with 11% acknowledging that more than half of the abdominal pain episodes were directly related to diet (28). Interestingly, these participants were more likely to have disorders Of The gut–brain axis (DGBAs) such as irritable bowel syndrome (IBS) (28). The mechanism(s) underlying food-related symptoms in most of these individuals remains poorly understood. As a result, dietary interventions often entail empiric restriction of various food groups.

The recent recognition of the gut microbiome as an important determinant of GI diseases has prompted investigations of the microbiome as a pivotal link between diet and host physiology. This seems logical, as both the host and the gut microbiome rely on diet for nutrition. Furthermore, both diet and the gut microbiome have been implicated in GI diseases ranging from functional disorders to inflammatory and neoplastic states. In this review, we focus the two best-studied conditions in the context of diet–microbiota interactions: IBS, the prototypical DGBA, and inflammatory bowel disease (IBD), a chronic inflammatory condition of the GI tract.

## 2. DIET-DERIVED BIOACTIVE METABOLITES ARE DETERMINED BY HOST–MICROBIAL COMETABOLISM AND THE CHEMICAL COMPOSITION OF THE DIET

The conventional wisdom is that most of the digestible components of food are rapidly assimilated via the large absorptive surface of the small intestine. The remaining indigestible components pass distally, where they serve as an energy source for the gut microbiota, leading to the generation of fermentation end products such as short-chain fatty acids (SCFAs). This concept, however, oversimplifies the process. Aside from its dependency on indigestible dietary components, the gut microbiota can derive nutrition from glycoproteins and polysaccharides in the mucus layer lining the host epithelial surfaces, especially in states of deprivation of microbiota-accessible carbohydrates, such as with low fiber intake. The utilization of available nutrients by individual members of the gut microbiota follows their nutritional hierarchy, along with cross-feeding patterns determined by the differential metabolic capacities of individual microbes. The cross-feeding patterns result in cooperative and competitive microbial networks that play an important role in determining the structure of the microbial community in the gut.

Nutrient utilization is not a matter of simple stoichiometry, whereby the total amount of a nutrient gets compartmentalized for the microbes and the host, but rather is a result of the meticulous amalgamation of their respective metabolic machineries. An alteration in microbial community structure can change the dynamics of such metabolic cooperation between the gut microbiome and the host. For instance, during homeostasis, efficient host uptake of amino acids in the small intestine makes them scarce for microbes; however, an overgrowth of amino acid–utilizing bacteria (e.g., Clostridia) can compete with the host, especially if dietary protein is limited. At the same time, increased availability of nutrients that are higher in the nutritional hierarchy (often mono- and disaccharides) reduces the utilization of amino acids by the same bacteria. In addition to bioavailability, regulatory signaling, such as by SCFAs or peptide YY, can affect the use of specific nutrients by the host. Therefore, metabolic end products often cannot be accurately inferred from the composition of a microbial community. In the following subsections, we highlight two examples (tryptophan and dietary fiber) of how differential nutrient utilization by the host and gut microbiota can affect host physiology.

### 2.1. Tryptophan-Derived Metabolites Differ in Host and Microbial Metabolism

**Figure 1:**
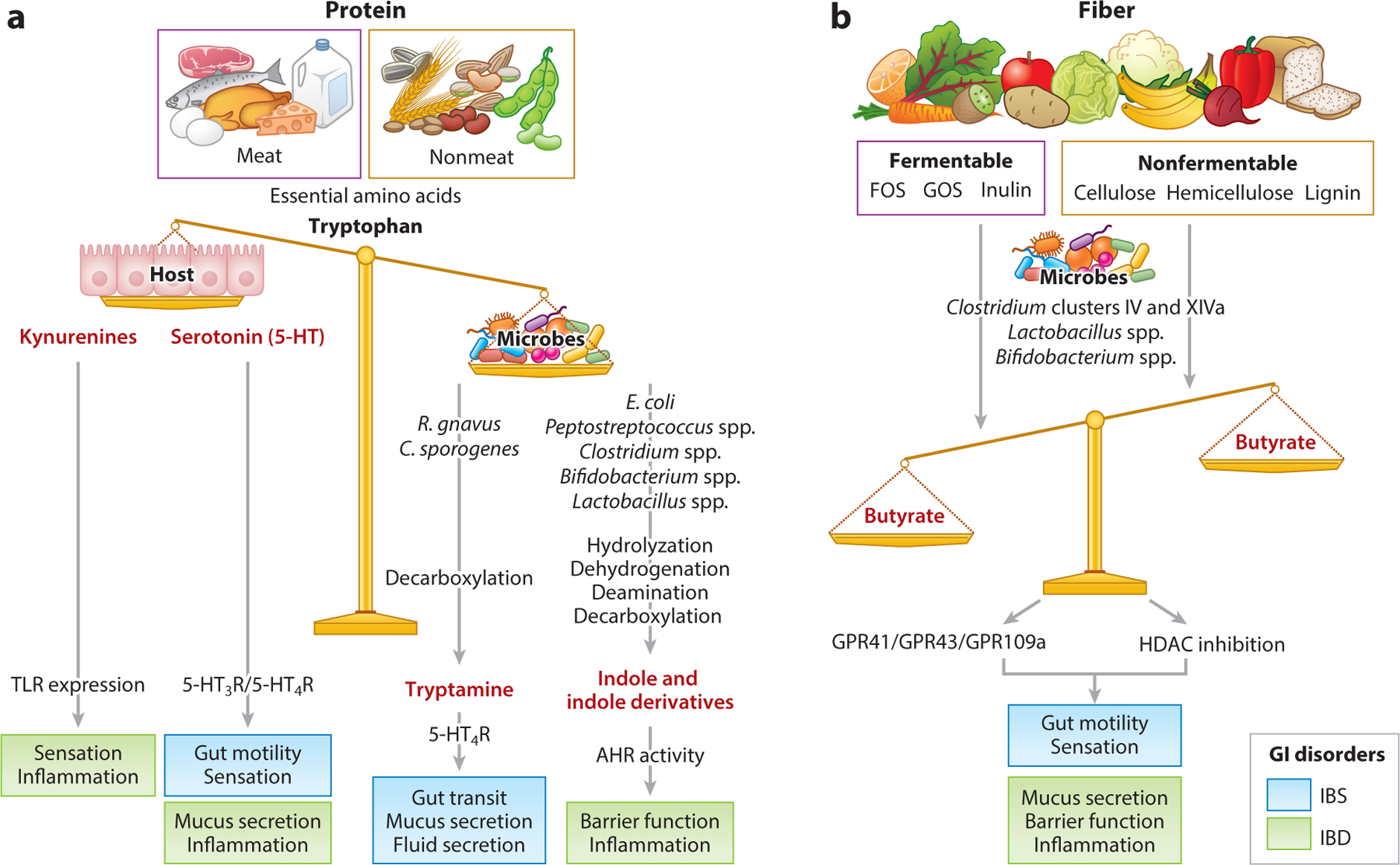
Diet-derived metabolites (*red text*) can alter multiple biologic pathways underlying diverse gastrointestinal (GI) diseases. (*a*) Dietary protein, both meat and nonmeat (e.g., grains, seeds, and nuts), includes varying levels of amino acids, such as tryptophan. Tryptophan can be metabolized by the host to produce kynurenines and serotonin (5-HT), which can alter gut physiology through the modulation of Toll-like receptors (TLRs) and serotonin receptors (5-HTRs), respectively. A small amount of tryptophan typically enters the colon, where it is synthesized by gut microbes. Gut microbiota can convert tryptophan to tryptamine or indole and indole derivatives via different metabolic pathways. Tryptamine increases intestinal secretion and mucus release from goblet cells by activating serotonin receptor 4 (5-HT_4_R), while indole and indole derivatives are ligands for aryl hydrocarbon receptor (AHR) and play an important role in regulating barrier function and immune responses. (*b*) Dietary fiber includes both fermentable [e.g., fructo-oligosaccharides (FOS), galacto-oligosaccharides (GOS), and inulin] and nonfermentable (e.g., cellulose, hemicellulose, and lignin) fiber. These are fermented into different short-chain fatty acids, such as butyrate and acetate, on the basis of the type of gut bacteria and the type of fiber. Butyrate acts on epithelial G protein–coupled receptors (GPCRs, e.g., GPR41, GPR43, and GPR109a) and as an epigenetic regulator by inhibiting histone deacetylase (HDAC) activity. Butyrate can increase serotonin synthesis, increase colonic contractility, alleviate visceral hypersensitivity, and augment the barrier. Physiological outcomes associated with irritable bowel syndrome (IBS) are shown in blue, and those associated with inflammatory bowel disease (IBD) are shown in green.

Tryptophan, an essential amino acid, is the precursor of the host neurotransmitter serotonin (5-HT; an important regulator of GI physiology) (Figure 1), as well as of microbial metabolites such as tryptamine and indole derivatives. The overall tryptophan pool depends largely on diet, with a small contribution from gut bacteria (120). Tryptophan is incorporated into proteins and utilized by the host to produce 5-HT (1–2%) and kynurenine (~95%) via distinct pathways (77) (Figure 1). Gut microbial members such as *Ruminococcus gnavus* and *Clostridium sporogenes* harbor tryptophan decarboxylase to convert tryptophan to tryptamine, which acts as an agonist of serotonin receptor 4 (5-HT_4_R) (149) (Figure 1). At the same time, bacteria such as *Bacteroides fragilis* and *Escherichia coli* harbor tryptophanase, which facilitates the production of indole and indole derivatives from tryptophan (88). Indole and its downstream products, such as indole acetic acid and indole propionic acid, can exert biological effects on host immune pathways by activating aryl hydrocarbon receptor (AHR) (82) (Figure 1). The levels of these bacterially produced tryptophan-derived bioactive metabolites depend on the gut microbiota composition, the extent and location of tryptophan utilization by gut bacteria, and the activity of genes involved in host tryptophan utilization.

### 2.2. Fermentation End Products Differ in Composition of Fiber and Gut Microbiota

A fiber-rich diet is considered beneficial because the gut microbiota ferments fiber to produce SCFAs such as butyrate, acetate, and propionate, which affect important aspects of host physiology including metabolism, cell turnover, and the immune system (145). However, human studies show significant interindividual variability in responses to fiber intake as well as differences based on fiber type. This is not surprising, as fiber is an umbrella term that includes diverse groups of carbohydrates with distinct linkages and molecular structures. Individual bacteria harbor genes allowing them to utilize carbohydrates with specific linkages and structures. The biological effects of dietary fiber will depend on the composition of the fiber, the potential of an individual’s gut microbiota to metabolize specific fibers, and the relative amounts of different fermentation end products (86, 117). A recent study (6) found that fructo-oligosaccharide (FOS) can worsen inflammation in patients with IBD, while its metabolism by gut bacteria reduces its inflammatory effects in IBD patients with active inflammation. Interestingly, FOS had an anti-inflammatory effect in healthy individuals. Thus, the inflammatory potential of FOS depends on gut microbial composition as well as on host disease status (6). The levels and types of SCFAs produced can vary according to the composition of fiber and the gut microbiota. The addition of inulin to the diet increased butyrate but decreased acetate production (86). In contrast, fecal butyrate levels were lower in subgroups of patients consuming the same amount of fiber, attributable to lower levels of butyrate-producing bacteria (Figure 1). These observations help explain the interindividual variability in responses to fiber observed in human studies.

### 2.3. Diet-Derived Metabolites Can Have Distinct Effects in Different Disease States

The concepts described above are relevant to a range of diseases, given that microbial metabolites exert pleiotropic effects on the host. Therefore, the same metabolite can affect multiple host functions, each of which may be relevant in different disease states. Tryptophan metabolites like tryptamine and 5-HT affect GI transit, which is relevant in DGBAs, while tryptamine and indole derivatives can alter mucus and the immune response, which has implications in IBD. Similarly, fermentation end products like butyrate can affect GI motility as well as epithelial barrier function, which are relevant in DGBAs and IBD, respectively (Figure 1).

## 3. DIET AS AN IMPORTANT DETERMINANT OF THE GUT MICROBIOME

Note that the above-described interactions are not static; instead, they change with changes in an individual’s gut microbiome. An important determinant of the gut microbiome is diet, which can have both long- and short-term effects on the gut microbiome. The long-term effects of a habitual diet are best demonstrated by the marked compositional alterations and reduced gut microbial diversity in individuals from industrialized countries when compared with those from agrarian societies (153). In a set of elegant experiments modeling these microbiomes in germ-free mice, Sonnenburg et al. (137) demonstrated that a low-fiber diet causes an incremental loss of gut microbial diversity in every successive generation, which is reversible in the early stages but results in extinction of specific taxa in subsequent generations that is unrecoverable by dietary intervention alone. This observation provides one explanation for the lower gut microbial diversity observed in Western populations and highlights how small changes can accumulate over generations; therefore, an individual’s microbial community structure likely reflects long-term dietary patterns in the population.

Short-term dietary alterations can also alter the gut microbiome. While these alterations are reversible to varying degrees, depending on the underlying resilience and adaptability of the community, short-term changes may explain in part the varying frequency and severity of symptoms in patients with chronic diseases. These short-term effects also underscore the potential for microbiota-directed dietary interventions as a therapeutic strategy.

## 4. IN SEARCH OF CULINARY CULPRITS

### 4.1. Diet Can Influence Symptoms of Irritable Bowel Syndrome via Microbiota-Independent and Microbiota-Driven Mechanisms

IBS is a common DGBA with a global prevalence of approximately 11.2% (49). It is diagnosed according to the presence of abdominal pain at least once a week, in association with defecation or a change in the frequency or form of stool, in the past 3 months with symptom onset within the past 6 months. IBS is categorized into diarrhea-predominant (IBS-D), constipation-predominant (IBS-C), mixed, and unclassified subtypes (53). Physiologic changes such as alterations in GI transit, secretion, sensation, immune activation, intestinal permeability, and the gut–brain axis underlie symptoms in IBS (12). Risk factors associated with IBS include host genetics, stress, psychiatric comorbidities, antibiotics, and early childhood experiences, but diet is most commonly identified by IBS patients as a potential culprit; population-based studies show that nearly 70% of IBS patients report perceived food intolerance (92, 101).

### 4.2. Mechanisms Linking Direct and Microbiota-Driven Effects of Diet with Irritable Bowel Syndrome Pathophysiology

The mechanisms by which diet can cause symptoms are still under investigation, but recent studies have begun to shed light on both microbiota-independent (Section 4.2.1) and microbiota-dependent mechanisms (Sections 4.2.2–4.2.6) underlying diet-driven symptoms in IBS.

#### 4.2.1. Diet and lipopolysaccharides.

Aguilera-Lizarraga et al. (1) found that direct injection of food antigens, such as gluten, wheat, milk, and soy, into the submucosa can trigger immune responses by activating mast cells in IBS patients but not in healthy subjects. They further showed that mast cell activation causes visceral pain and increases intestinal permeability via histamine-stimulated sensitization of visceral neurons. While this study demonstrated a microbiota-independent mechanism, other studies have found that diets high in fermentable oligo-, di-, and monosaccharides and polyols (FODMAP) can also activate mast cells via the Toll-like receptor 4 (TLR4) pathway, implicating the involvement of gut microbiota (132). Lipopolysaccharides (LPS), a group of heterogeneous cell wall components of gut bacteria which act as ligands for TLR4, are also increased among individuals consuming a high-fat Western diet (115) or a high-FODMAP diet. Apart from its role in mast cell activation, LPS in different forms promotes the survival of enteric neurons (4) and increases smooth muscle contractility (102), suggesting that differences in LPS concentration or structure may drive different host responses. Serum levels of microbial products such as LPS and flagellin, which are affected by diet, have been reported to be significantly elevated in patients with IBS-D (36).

#### 4.2.2. Metabolites derived from microbial fermentation of fiber.

In addition to microbial cell wall components, metabolic end products resulting from host–microbial metabolism of dietary ingredients can drive GI symptoms through their effect on the underlying GI physiology. SCFAs such as acetate, propionate, and butyrate are produced by specific gut microbiota members, and their levels depend on both microbiota composition and dietary fiber intake. Butyrate is a pleiotropic metabolite that can directly signal via G protein–coupled receptors (GPCRs) and alter transcriptional responses via epigenetic modulation (30). Butyrate can alter 5-HT synthesis in enterochromaffin cells in a concentration-dependent manner (122), increase colonic contractility through direct effects on the enteric neuromuscular apparatus, augment the intestinal epithelial barrier, and regulate visceral hypersensitivity via interactions with enteric glia (76). Intracolonic acetate, on the other hand, enhances sensitivity to colorectal distention (150). The specific effects likely depend on host health and the overall metabolite milieu in the gut.

#### 4.2.3. Tryptophan-derived microbial metabolites.

Diet, host mucus, and microbial production are all key sources of amino acids in the gut.A longitudinal study reported that try ptophan and tryptamine levels, but not indole derivatives, were higher in patients with IBS-D than in healthy individuals, despite similar dietary protein intake (95). This difference could be a result of increased tryptophan production and conversion to tryptamine by gut microbiota or, alternatively, decreased utilization by the host. Tryptamine activates 5-HT_4_R present on the enterocytes and increases intestinal fluid secretion. Another study found no differences among IBS patients and healthy subjects in 5-HT_4_R expression or response of colonic tissue to tryptamine, suggesting that higher tryptamine levels are likely an important driver of diarrhea (13).

#### 4.2.4. Gluten.

Other diet- and microbiota-driven pathways have been described in IBS-D. Gluten intolerance, which is frequently reported among IBS-D patients in the absence of celiac disease, appears to depend partly on the host genotype (7). IBS-D patients negative for *HLA-DQ2*/*HLA-DQ8* (permissive of but not diagnostic for celiac disease) have been reported to experience a greater reduction in abdominal distention following a gluten-free diet in comparison to their negative counterparts. While this study (7) did not specifically investigate the role of the gut microbiota, other studies have found that the gut microbiota can differentially affect gluten digestion and immunogenicity (e.g., 17). The specific mechanism underlying the effect of gluten in IBS-D still needs to be determined.

#### 4.2.5. Microbial bile acid metabolism.

Bile acids (BAs) are synthesized in the liver, stored in the gallbladder, and used for lipid emulsification for rapid absorption in the small intestine upon release. Dietary fat and turmeric are important stimuli for the release of primary BAs into the small intestine (32). The two primary BAs in humans—chenodeoxycholic acid (CDCA) and cholic acid (CA)—are conjugated with glycine or taurine (at a ratio of three to one). Nearly 95% of the primary BAs are reabsorbed in the distal small intestine. The remaining primary BAs entering the colon are deconjugated, dehydroxylated, and epimerized to secondary BAs—lithocholic acid from CDCA and deoxycholic acid from CA—by gut microbes (100). Primary BAs such as CDCA increase colonic secretion via chloride channels and lower rectal sensory thresholds in healthy individuals (10, 111). In a rodent model, they affected visceral sensitivity through the activation of nuclear receptor farnesoid X receptor, release of nerve growth factor, and downstream expression of transient receptor potential vanilloid 1 in the dorsal root ganglia (89). Patients with IBS-D are likely to have higher levels of fecal BAs, attributable to BA malabsorption and/or a decrease in gut microbiota–driven conversion to secondary BAs (35). Therefore, a high-fat diet can alter GI physiology either directly, by regulating BA release, or indirectly, through microbial metabolism of BAs (154).

#### 4.2.6. Microbial β-glucuronidases.

A recent study found that patients with postinfectious IBS-D have lower levels of bacteria-encoded β-glucuronidases, which can deconjugate bilirubin (39). These patients had higher levels of conjugated bilirubin, which led to decreased inhibition of host proteases and increased intestinal permeability, contributing to the visceral hypersensitivity observed in IBS-D patients. Several additional mechanisms underlying microbiota-driven visceral hypersensitivity have been identified in preclinical models; these include neurotransmitter/peptide-mediated hyperalgesia (e.g., 5-HT, calcitonin gene–related peptide, substance P) (40, 139), altered neuroreceptor signaling [e.g.,5-HT receptors, GABAergic signaling, GPCRs including protease-activated and cannabinoid receptors (reviewed in 3)] (20, 40, 155), and guanylate cyclase C signaling (21).

### 4.3. Diet Is an Important Determinant of Symptoms and Disease Activity in Inflammatory Bowel Disease

IBD is an idiopathic, chronic, debilitating, inflammatory disorder of the GI tract, encompassing two conditions—Crohn’s disease (CD) and ulcerative colitis (UC). While CD manifests as a patchy transmural inflammation that can be scattered throughout the GI tract, UC is a continuous mucosal inflammation of the colon. Both disorders result from an uncontrolled inflammatory response to gut microbial cues, in the milieu of interacting environmental, genetic, and immunological factors. Epidemiologically, IBD—which used to be a disease of the Western world, with the highest prevalence in European and North American nations (0.5% in the USA)—has made a great shift to the east since the 1990s, with incidences rising rapidly in newly industrialized countries in Africa, Asia (incidence in India of 9.3 cases per 100,000 person years and in China of 3.3 per 100,000 person years), and South America. This shift has been attributed to environmental factors arising from the rapid westernization and industrialization of these societies (73, 75, 107).

These epidemiological transitions have coincided with global shifts in dietary patterns, including the introduction of packaged and processed foods; wide acceptance and usage of food additives, preservatives, and antibiotics; and the promotion of fast-food chains, accompanied by the diminishment of region-specific, local-food diets. The role of diet as one of the key environmental factors shaping IBD risk is demonstrated by studies on migrant epidemiology reporting an enhanced prevalence of IBD in populations migrating from low-incidence areas to high-incidence regions (33). Along with the more pronounced global east–west epidemiological patterning of IBD, a more subtle north–south prevalence disparity is evident in France and Spain. A higher IBD load is observed in the northern parts of these nations, where individuals consume more butter, potatoes, ham, cheese, sausage, and beer, whereas individuals in the southern regions follow a Mediterranean diet (MD), composed mainly of olives, fresh fruits and vegetables, wine, and seafood (22, 116).

### 4.4. Mechanisms Linking Direct and Microbiota-Driven Effects of Diet with Inflammatory Bowel Disease Pathophysiology

Dietary components can drive pathophysiology of IBD both directly and following their transformation by gut microbiota.

#### 4.4.1. Animal protein and trimethylamine-*N*-oxide.

**Figure 2:**
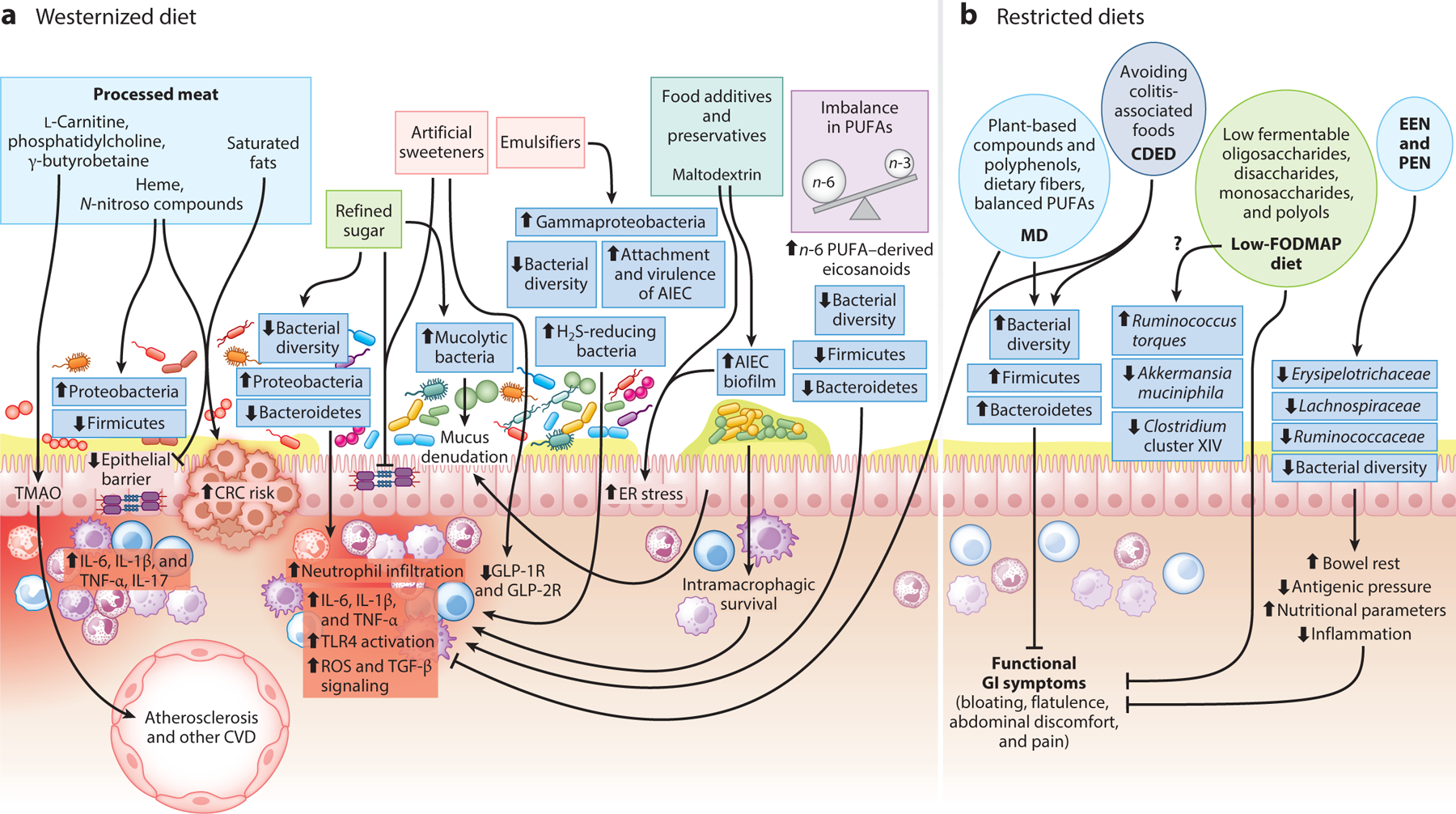
Role of diet in the pathogenesis and prevention of inflammatory bowel disease (IBD). (*a*) A Westernized diet is rich in ultraprocessed foods, processed red meat, foods with high refined sugar content, artificial sweeteners, food additives, preservatives, and emulsifiers. Processed red meat is rich in l-carnitine, phosphatidylcholine, and γ-butyrobetaine, which are converted to trimethylamine-*N*-oxide (TMAO) by the action of gut microbial and host liver enzymes. TMAO has been implicated in enhancing the risk of cardiovascular disease (CVD). Additionally, an abundance of heme and *N*-nitroso compounds, along with saturated fats, promotes gut dysbiosis, hampers epithelial barrier integrity, enhances the release of inflammatory cytokines, and increases the risk of colorectal cancer (CRC). Refined sugars and artificial sweeteners mediate their downstream inflammatory effects by decreasing bacterial diversity, enhancing Proteobacteria and mucolytic bacteria, and reducing beneficial Firmicutes and Bacteroidetes. Artificial sweeteners such as sucralose and acesulfame potassium also mediate their inflammatory effects through inhibition of glucagon-like peptide 1 and 2 receptors (GLP-1R and GLP-2R). Emulsifiers such as polysorbate 80 and carrageenan mediate gut inflammation by promoting gut dysbiosis (enhancement of Gammaproteobacteria, a class of sulfide-reducing bacterial genera) and promote the attachment and virulence of adherent-invasive *Escherichia coli* (AIEC). Similarly, the common food additive maltodextrin promotes AIEC attachment and biofilm formation. An imbalanced (versus the ideal one-to-one) ratio of *n*-6 to *n*-3 polyunsaturated fatty acids (PUFAs), through the elevation of *n*-6 PUFAs, is also a characteristic feature of sedentary diet regimes and mediates gut inflammation and dysbiosis, characterized by reduced bacterial diversity, and reduces the numbers of beneficial gut bacterial members of the phyla Firmicutes and Bacteroidetes. (*b*) Dietary regimes that have been actively explored for the alleviation of IBD symptoms include the Mediterranean diet (MD) and the Crohn’s disease–exclusion diet (CDED), both of which exert their anti-inflammatory effects through the enrichment of gut bacterial diversity and beneficial bacterial genera of the phyla Firmicutes and Bacteroidetes. MD is rich in dietary fiber and *n*-3 PUFAs, which are widely accepted to reduce neutrophil infiltration and expression of inflammatory cytokines in the gut. Low-FODMAP (fermentable oligo-, di-, and monosaccharides and polyols) diets, partial enteral nutrition (PEN), and exclusive enteral nutrition (EEN) alleviate gut inflammation, improve nutritional status, and relieve functional gastrointestinal (GI) symptoms as a result of their simpler constitution, leading to lower antigenic pressure and subsequent bowel rest. However, their administration has been linked to a reduction in both gut microbial diversity and beneficial gut microbial members. Therefore, the impact of their long-term administration, their therapeutic efficiencies, and the underlying mechanisms must be assessed in robust clinical trials.

The complex interactions among dietary macronutrients, micronutrients, additives, and caloric content; host immunity; genetics; and the gut microbiome are likely important determinants of the risk and clinical course of IBD (Figure 2). A recent large prospective cohort of 125,445 participants found an association between a Western diet, consisting of animal protein such as red meat, poultry, and processed meats, and an increased likelihood of UC development (37). These IBD-exacerbating effects of red meat were also highlighted in two additional study cohorts—the European Prospective Investigation into Cancer and Nutrition cohort (142), demonstrating that an increased linoleic acid intake associated with red meat consumption increases the risk of developing UC more than twofold, and a large French prospective questionnaire study (68). Interestingly, the consumption of processed red meat, but not unprocessed red meat, poultry, or fish, has recently been significantly correlated with increased mortality in patients with CD (23). Red meat is composed mainly of protein, fat, and heme. Elevated protein, fat, and dietary heme alter the gut microbiota composition, which, in turn, negatively affects epithelial cell turnover and gut barrier integrity and elevates intestinal inflammation (Figure 2). Red meat is rich in l-carnitine, phosphatidylcholine, and γ-butyrobetaine, which, through gut microbial metabolism, are converted to trimethylamine, a precursor for the formation of trimethylamine-*N*-oxide (TMAO) by the host liver flavin-containing monooxygenases (133, 143). Animal data as well as human epidemiological studies show a strong positive association between TMAO and inflammation (5, 45), cardiovascular diseases, colorectal cancer (67), and mortality (69).

#### 4.4.2. Processed foods.

The Western diet is rich in (ultra)processed foods, a category encompassing a wide variety of food groups including meat, starchy snacks, dairy, legumes, fruits, and vegetables. Unlike traditional dietary regimes, the Western diet is enriched in simple refined carbohydrates, saturated fats, and processed and industrialized foods, and is lower in fresh fruits and vegetables, legumes, whole cereals, and dietary fiber. Studies have reported detrimental effects of the Western diet on human health and have linked it with obesity, diabetes, IBD, chronic kidney diseases, and other lifestyle-associated disorders. (Ultra)processing of food items aims to enhance their shelf life, palatability, and convenience of storage and distribution, and it involves the incorporation of many nonnatural ingredients and additives such as artificial flavors, stabilizers, preservatives, and emulsifiers. A recent study on a large prospective cohort (116,087 adults) from 21 low-, middle-, and high-income countries across seven geographical regions found that higher intake of ultraprocessed foods was positively correlated with a risk of IBD; however, intake of unprocessed white meat, red meat, dairy, starch, fruits, and vegetables was not associated with the incidence of IBD (105).

#### 4.4.3. Dietary sugars and artificial sweeteners.

Studies have found a significant positive correlation between IBD risk and consumption of nonalcoholic sugary soft drinks. Two recent meta-analyses compiling observational studies on beverage intake and IBD risk mirrored these findings, demonstrating that high intake of soft drinks is positively associated with IBD risk (78, 109). Experiments have found that high dietary sugar is associated with inflammation induction and gut dysbiosis. Interestingly, a questionnaire-based study (121) comparing the dietary pattern of patients with IBD with that of the healthy population showed higher soft drink consumption in patients with IBD (Figure 2).

Artificial sweeteners such as aspartame, saccharine, acesulfame potassium, and sucralose have gained wide acceptance for imparting sweetness without adding extra calories. However, animal studies and trials in healthy human subjects reported that these nonnutritive sweeteners reduce gut microbial diversity (24, 46), perpetuate gut inflammation (54), alter the gut microbiota by enhancing members of Proteobacteria and reducing the representation of beneficial microbial members [*Ruminococcaceae*, *Lachnospiraceae*, and *Clostridium* cluster XIVa (144)], and compromise gut barrier integrity, especially through reduced expression of glucagon-like peptide 1 and 2 receptors (57) (Figure 2). Similarly, synthetic emulsifiers such as polysorbate 80 and carboxymethyl cellulose, which are used as additives to enhance texture and boost shelf life, have been widely implicated in animal studies as causing gut dysbiosis and promoting chronic inflammation (104). In vitro studies utilizing Peyer’s patches from CD patients showed increased translocation of bacteria such as *E. coli* across M cells and Peyer’s patches, along with enhanced bacterial adherence to the intestinal epithelium and increased translocation and infiltration of these bacteria between intestinal villi (124).

#### 4.4.4. Food additives.

Maltodextrin (E1400), another important food additive that is used as a thickener in processed foods, exacerbated intestinal inflammation in a dose-dependent manner in a murine model of colitis, through induction of endoplasmic reticulum stress and alterations of the mucus layer (83). Reports in mouse models also indicate that maltodextrin favors biofilm formation through CD-associated adherent-invasive *E. coli* through modulation of bacterial gene expression (108).

The deleterious effects are enhanced by preservatives in processed foods. Sodium benzoate (E211), sodium nitrite (E250), and potassium sorbate (E202), three of the most commonly used preservatives, reduce gut microbial diversity, with increased representation of Proteobacteria and reduced Clostridiales in a human gut microbiota–associated mouse model, at exposure levels typical of European populations (65). Even though human and animal studies have provided mechanistic insights into the negative effects of these nonnutritive dietary additives on gut dysbiosis and intestinal health, randomized controlled trials in humans evaluating the impact of these sweeteners in IBD cohorts are lacking.

#### 4.4.5. Polyunsaturated fatty acids.

A prominent feature of the Western diet is the significantly greater contribution of energy from *n*-6 polyunsaturated fatty acids (PUFAs) versus *n*-3 PUFAs. A large, prospective, epidemiological study by Tjonneland et al. (142), based on food frequency questionnaires from more than 200,000 participants across multiple centers, showed a significant association between intake of the *n*-6 PUFA linoleic acid and increased risk of UC. A systematic literature review by Hou et al. (64) reported an increased risk of developing UC with a high intake of total fat, *n*-6 PUFAs, and meat, as well as an increased risk of CD with a high intake of saturated fats, *n*-6 PUFAs, and meat. While the major dietary *n*-3 PUFAs, namely eicosapentaenoic acid (EPA) and docosahexaenoic acid, and their downstream eicosanoids have anti-inflammatory properties, *n*-6 PUFAs such as arachidonic acid (AA) and their eicosanoids, such as prostaglandins, thromboxanes, leukotrienes, hydroxyeicosatetraenoic acid, lipoxins, and epoxyeicosatrienoic acid, demonstrate strong proinflammatory activity in IBD. These mediators potentiate neutrophil chemotaxis; enhanced vascular permeability; and production of inflammatory cytokines such as tumor necrosis factor (TNF)-α, interleukin (IL)-1β, IL-6, and IL-8 (Figure 2). Interestingly, the metabolism of these fatty acid mediators is itself altered in inflamed mucosa, with higher *n*-6 PUFA AA, lower *n*-3 PUFA EPA, and a higher AA-to-EPA ratio, suggesting that fatty acid metabolism is a vicious perpetuator of inflammation in IBD (112).

Recent animal studies and human trials have linked dietary *n*-6 PUFAs to gut microbial dysbiosis. Miao et al. (97) reported that higher levels of γ-linolenic acid are significantly associated with higher incidence of type 2 diabetes; reduced gut microbial diversity; and reduced beneficial microbial genera such as *Prevotella*, *Odoribacter*, *Faecalibacterium*, *Paraprevotella*, *Blautia*, and *Butyrivibrio*, as well as members of Clostridiales, *Rikenellaceae*, and *Coriobacteriaceae*. Mice supplemented with an *n*-6 high-fat diet at the weaning stage demonstrated an increase in the number of colonic inflammatory and hyperplastic lesions during adulthood, coupled with a marked reduction in members of Firmicutes, Clostridia, and *Lachnospiraceae* and an enhancement in the representation of proinflammatory *Mucispirillum schaedleri* and *Lactobacillus marinus* (128). Similar effects of *n*-6 supplementation were observed in an aged mouse model, where an *n*-6 high-fat diet reduced beneficial members of Firmicutes and Bacteroidetes and caused gut inflammation. The observed gut dysbiosis was reversed by fish oil supplementation (51).

## 5. DIETARY INTERVENTIONS IN GASTROINTESTINAL DISEASE

Patients’ perceived intolerance to dietary components, and the role of these components in the pathophysiology of GI diseases such as IBS and IBD, has made diet a frequent target of therapeutic approaches. However, dietary strategies lack specificity, and similar approaches have been used across GI diseases with distinct pathophysiologies, like IBS and IBD. These strategies fall into several different categories, the most common being restricting, altering, or supplementing nutrients.

### 5.1. Dietary Restrictions

The most common form of dietary modification is restriction of nutrients that are considered to be important drivers of disease pathophysiology.

#### 5.1.1. Reducing fiber-rich foods.

A common strategy based on the rationale that increased gas production underlies bloating is to reduce foods rich in fermentable fiber. However, this rationale is not supported by current evidence, which suggests that visceral hypersensitivity and decreased movement of gas, rather than increased gas production, are what underlie symptoms of bloating. The benefit of this strategy, if any, is often short-lived. In fact, a systematic review (42) found that long-chain, moderately fermentable soluble dietary fiber, like psyllium, improves symptoms in IBS; therefore, restricting fiber may worsen symptoms over the long term. In contrast, a low-fiber diet is recommended for patients with IBD when there is stricturing disease to prevent episodes of small bowel obstruction. The response to fiber supplementation or restriction is likely dependent on the type of fiber (80), the underlying disease state, and the gut microbiota composition, making it difficult to suggest a one-size-fits-all approach. Therefore, treatment needs to be individualized for every patient.

#### 5.1.2. Low-FODMAP and gluten-free diets.

One of the most common dietary interventions in IBS is reducing FODMAP (typically poorly absorbed short-chain carbohydrates including fructose, lactose, polyols, fructans, and galacto-oligosaccharides) for 12 weeks, followed by slow reintroduction of the food groups. This intervention is partly based on the notion that FODMAP increase osmotic load and generate higher levels of hydrogen, resulting in luminal distention. A pivotal study by Halmos et al. (56) in Australian subjects with IBS showed significant improvement in symptoms in comparison to a Western diet. A recent meta-analysis (34), which included seven randomized controlled studies of 397 patients, showed that a low-FODMAP diet reduced global symptoms compared with control interventions. However, the three randomized controlled trials within this meta-analysis, which compared a low-FODMAP diet with rigorous control diets, had the least heterogeneity among studies and the least magnitude of effect. As a result, the authors concluded that while a low-FODMAP diet can benefit IBS patients, the overall quality of data was very low. This finding suggests that several different dietary interventions improve IBS symptoms, and it would be helpful to find common elements among them. Interestingly, a study in healthy subjects found no reduction in colonic volume with a low-FODMAP diet (135), suggesting that an alternate mechanism may underlie the improvement in symptoms. An important off-target result of a low-FODMAP diet is its deleterious effect on the gut microbiota; the long-term implications of these changes remain unclear. There is also concern that patients may develop avoidant/restrictive food intake disorders, especially because the reintroduction of foods is particularly difficult.

The above-described meta-analysis found no significant benefit of a gluten-free diet in IBS patients. However, as mentioned above, the effect may be dependent on host genotype or other host/environmental factors. Gluten is found mainly in wheat, barley, and rye, which are part of a high-FODMAP diet; therefore, the improvement observed in subsets of patients may also be a result of FODMAP restriction rather than of gluten alone (94). A recent review (62) showed a high prevalence of nonceliac gluten sensitivity in IBD patients; however, there is scant evidence to support a gluten-free diet in these patients. Preclinical studies (96, 156) found an improvement in inflammation and permeability with a gluten-free diet, but there is a lack of high-quality prospective studies in human subjects. The emerging literature on microbial degradation of gluten has implications for both IBD and celiac disease and is an important area for future investigation.

#### 5.1.3. Exclusive or partial enteral nutrition.

Exclusive enteral nutrition (EEN) has been accepted as a first-line dietary intervention for pediatric patients with CD. EEN is based on an exclusively elemental (liquid) diet complete with all essential macronutrients and micronutrients, administered exclusively instead of solids and fluids for 8–12 weeks (146). Many studies have reported that the efficacy of EEN in inducing remission in pediatric patients with mild to moderate CD is comparable to that of corticosteroids (e.g., 63). For instance, in independent Australian and Spanish trials, EEN supplementation for 8 weeks resulted in clinical remission in 84% and 80% of subjects, respectively (106). EEN is also efficacious in perioperative adult patients with CD. A meta-analysis of two prospective cohort studies (151) showed a significant reduction in postoperative complications between patients who received preoperative EEN (22%) versus those who did not. Although limited, other studies have described the benefits of EEN in the management of penetrating CD (152), stricturing CD (61), and extraintestinal CD (103).

Mechanistically, EEN likely exerts its effects through compositional and functional alterations in gut microbiota. Even though it paradoxically reduces gut microbial diversity and abundance of taxa often considered beneficial [members of genera *Faecalibacterium*, *Ruminococcus*, and *Bifidobacterium* and other members of families *Erysipelotrichaceae*, *Lachnospiraceae*, and *Ruminococcaceae* (38)], it enhances the functional capacity of the gut microbiota on the basis of changes in metabolites (93). Due to the simple composition of EEN, a reduction in antigenic pressure and bowel rest may also be important modes of action. Additionally, active ingredients in the EEN formula improve nutritional parameters and may exert anti-inflammatory effects on the intestinal epithelium. EEN is used in adults as a second- or third-line treatment, with corticosteroids as the primary induction therapy, as these are more effective than EEN for induction of clinical remission. However, clinical trials assessing the efficacy of EEN in adult patients with CD have been limited by low sample sizes and higher rates of noncompliance with the diet.

Partial enteral nutrition (PEN), which involves supplementation of half of a patient’s caloric requirement as enteral nutrition along with a whole-food diet, is beneficial for long-term maintenance of remission in patients with CD. Unrestricted PEN in combination with an elemental formula had limited efficacy in a pediatric CD cohort published in 2006 (70); as a result, researchers conceived of the need for a CD- and UC-specific exclusion diet that excludes certain detrimental food items. The Crohn’s disease exclusion diet (CDED), combined with PEN, is a whole-food diet regime designed to reduce exposure to dietary components and foods associated with deleterious changes in gut microbiota (such as expansion of Proteobacteria), compromised barrier integrity, and inflammation in the GI tract. CDED is a multistage, high-protein, low-fat diet consisting of a 12-week induction phase, in which the patient consumes specific foods, followed by a 6-week maintenance phase, in which additional food items are introduced. CDED excludes processed foods and incorporates beneficial fibers, coupled with a liquid formula to meet the patient’s energy needs. A prospective study reported that CDED plus PEN were better tolerated and more effective in a CD cohort when compared with EEN, and that 75% of patients on CDED plus PEN underwent steroid-free clinical remission (85).

### 5.2. Dietary Modification Toward a Mediterranean Diet

MD is rich in fruits, vegetables, bread, cereals, beans, nuts, and virgin olive oil, along with moderate amounts of dairy, fish, and meat. Table 1 lists human trials assessing the efficacy of MD in IBD. A recent prospective, randomized study including 100 adolescent IBD patients with mild to moderate disease compared the efficacy of MD with that of the regular diet, showing a significant decrease in clinical scores on the Pediatric Crohn’s Disease Activity Index and the Pediatric Ulcerative Colitis Activity Index as well as lower levels of inflammatory markers, such as serum C-reactive protein, calprotectin, TNF-α, IL-17, IL-12, and IL-13 (41). A recent clinical trial by Chicco et al. (26) also observed beneficial effects of MD in IBD. This study involved 142 IBD patients (84 UC and 58 CD) following MD for 6 months. Diet adherence significantly improved body mass index and waist circumference and led to a marked reduction in liver steatosis– and malnutrition-related parameters (26). Similar beneficial effects of MD on IBD were demonstrated by the DINE-CD study (87), in which 40% of patients with mild to moderate CD underwent remission following 6–12 weeks of MD. MD has been associated with beneficial gut microbial profiles, specifically, with enrichment of dietary fiber metabolizers such as *Faecalibacterium prausnitzii*, *Bacteroides cellulosilyticus*, and *Prevotella*, along with other microbes involved in degradation of plant polysaccharide and production of SCFAs and secondary BAs (31).

MD is rich in *n*-3 PUFAs, such that the *n*-3 and *n*-6 PUFAs strike a perfect balance. Wiese et al. (148) demonstrated the positive effects of EPA and other PUFAs in a prospective UC cohort, where gut inflammatory cytokine levels correlated inversely with PUFA, EPA, and docosapentaenoic acid. A recent study by Scaioli et al. (127) found that 2 g/day of EPA reduced fecal calprotectin levels (100-point reduction) and maintained clinical remission in UC patients (endpoint achieved in 76.6% of the participants on EPA versus 50% in placebo). Costea et al. (29) associated single-nucleotide polymorphisms across three crucial genes (*CYP4F3*, *FADS1*, and *FADS2*) involved in *n*-3 fatty acid metabolism with an increased risk of CD, implicating an additional, genetic dimension of diet-associated regulation of IBD. *n*-3 PUFAs likely exert their anti-inflammatory effects through downstream lipid mediators such as resolvins, protectins, and maresins, which can counter IBD-associated inflammation (129). Mechanistically, *n*-3 PUFAs have been found to (*a*) decrease chemotaxis of neutrophils and monocytes toward various chemoattractants (52, 140); (*b*) suppress TLR4 expression and NOD2 signaling by blocking the release of nuclear factor κB from mitogen-activated protein kinase (2); (*c*) inhibit NLRP3 inflammasome activation and subsequently hamper the release of proinflammatory cytokines (131); and (*d*) increase the abundance of the butyrate-producing bacterial genera *Bifidobacterium*, *Roseburia*, and *Lactobacillus*, as well as members of the family *Lachnospiraceae* (147).

MD leads to increased production of SCFAs by the gut microbiota as a result of higher levels of fermentable carbohydrates. Zito et al. (157) found that MD may improve bloating and abdominal pain in IBS patients who adhere to the diet. A recent review comparing the low-FODMAP diet and MD found reciprocal effects on the gut microbiota (74); the long-term implications of such changes still need to be determined.

### 5.3. Supplementing Diet with Fiber or with Pro-, Pre-, or Synbiotics

This section focuses on the effect of nutrients such as in fiber, prebiotics, and synbiotics that promote bacterial communities with a beneficial effect on health, as well as live biotherapeutic products (probiotics) shown to improve host function.

#### 5.3.1. Fiber, prebiotics, and synbiotics.

A meta-analysis of 14 randomized controlled studies including 906 IBS patients found a significant improvement in symptoms with soluble fiber but not bran (99). Most of these studies used fiber supplementation; few of them modified the diet to increase fiber intake. Although several studies have investigated prebiotics and synbiotics, there are insufficient data to make recommendations (27). A randomized, parallel, double-blind study in IBS patients comparing the effects of an MD-type diet and a prebiotic supplement (β-galacto-oligosaccharide) with a low-FODMAP diet and a placebo xylose supplement found a similar improvement in symptoms in both groups, but a more favorable gut microbiota profile with prebiotic supplementation (66) (Table 2). This finding highlights the potential for dietary modification and supplementation as an alternative to restrictive dietary practices in the management of GI diseases.

Preclinical models of IBD have found that a high-fiber (predominantly psyllium), low-protein diet augments intestinal barrier function and reduces inflammation (91). Thus, soluble fiber appears to benefit both patients with IBS and those with IBD. A recent meta-analysis found a linear dose-dependent relationship between dietary fiber intake and CD risk, with a 13% depreciation in CD risk for every 10 g/day increment in fiber intake (90). Food- and supplement-based fiber and prebiotic intervention studies have reported encouraging results with a fiber-enriched semivegetarian diet (25), *Plantago ovata* seeds (47), oat bran (55), and germinated barley foodstuff (43) in the maintenance of remission and significant improvement in GI symptoms (abdominal pain and reflux). Similar results have been reported in active disease cohorts receiving supplementation of FOS (15 g/day for 3–4 weeks) (11), oligofructose-enriched inulin (10 g twice per day for 4 weeks) (71), whole wheat bran (0.5 cup/day for 4 weeks) (15), inulin-type fructan (7.5 g/day for 9 weeks) (19), and germinated barley foodstuff (72), specifically, a significant reduction in disease activity (according to the Harvey–Bradshaw index) and an improvement in quality of life. A systematic review and meta-analysis assessing the effects of fiber intake on the gut microbiome composition showed that dietary fiber is associated with significantly higher abundances of *Bifidobacterium* spp. and *Lactobacillus* spp., as well as with higher levels of fecal butyrate, when compared with a placebo/low-fiber diet (136). These studies were conducted largely in adult IBD patients, and the benefit for the pediatric population remains unclear. Note that fiber is used broadly to include a range of complex carbohydrates (including prebiotics). As mentioned above, the effects of fiber will likely differ on the basis of the carbohydrate structure, health status, and gut microbiota composition.

Some studies have demonstrated a benefit of synbiotics in adult patients with UC. A 4-week supplementation of a combination of *Bifidobacterium longum* and oligofructose-enriched inulin was associated with sigmoidoscopic and histopathological improvements and reduced the expression of inflammatory cytokines (TNF-α and IL-1β) in comparison to a placebo group (48). Altun et al. (3) reported similar results in a randomized controlled trial involving 8-week supplementation of a symbiotic cocktail composed of *Enterococcus faecium*, *Lactobacillus plantarum*, *Lactobacillus acidophilus*, *Streptococcus thermophilus*, *Bifidobacterium lactis*, *B. longum*, and FOS. A meta-analysis assessing the efficacy of synbiotics in UC revealed encouraging results about the safety and efficacy of synbiotics as a therapeutic option for UC (126). However, the beneficial effects of synbiotics are questionable in pediatric IBD cohorts and in patients with CD, due to reports of poor efficacy and tolerability (58).

#### 5.3.2. Probiotics.

While probiotics have failed to show efficacy in IBS or IBD (118), randomized controlled trials have found that they can mitigate the deleterious effects of dietary restriction on gut microbiota. The potential physiologic implications of such effects remain unclear (138).

## 6. ROLE OF GUT MICROBIOTA IN GENETICALLY DRIVEN DISEASES

### 6.1. Lactose Intolerance

Lactose consumed via milk and milk products is digested in the small intestine via the hydrolytic action of a brush border enzyme called lactase phlorizin hydrolase (LPH). The expression of this enzyme declines as an infant shifts from a largely milk-based diet to other sources of nutrition. Most instances of lactose intolerance (e.g., flatulence, bloating, abdominal discomfort/pain, and diarrhea) can be attributed to adult-onset hypolactasia resulting from decreased expression of LPH. Genetic polymorphisms resulting in inactive or dysfunctional LPH and GI disorders that damage the intestinal epithelium can lower LPH production (141). Undigested lactose exerts osmotic pressure in the lumen, triggering diarrhea and abdominal discomfort, and fermentation of lactose by microbial lactase (β-galactosidase) results in increased flatulence (59, 60). Yogurt appears to be an exception, as bacteria commonly used for the production of yogurt (*Lactobacillus delbrueckii* subsp. *bulgaricus* and *S. thermophilus*) produce high levels of β-galactosidase, which depletes lactose both in the yogurt and in the small intestine (79). Therefore, the presence of these bacteria in the small intestine, or their consumption as probiotics or yogurt, may reduce symptoms to varying degrees.

### 6.2. Celiac Disease

Celiac disease is an autoimmune disease whereby patients mount an immune response to gluten in the small intestine, resulting in diarrhea, abdominal pain, and malabsorption that in turn cause weight loss and malnutrition. Celiac disease has been associated with other autoimmune and genetic disorders, including primary biliary cirrhosis, type 1 diabetes, ataxia, Addison’s disease, and Down syndrome (84, 130). Gluten comprises small peptides such as gliadin and glutenin and is the key component of the dietary grains wheat, barley, and rye. Gluten is deaminated in the lamina propria by tissue transglutaminase and presented as antigens by the major histocompatibility class II molecules encoded by the specific haplotypes *HLA-DQ2* and *HLA-DQ8*, initiating a cascade of proinflammatory Th2 response that in turn causes intestinal enteropathy. The presence of this haplotype is necessary but insufficient to cause disease, and other environmental cues are likely needed (16, 130). The threshold of gluten exposure that can stimulate responses is below 10 mg; therefore, the primary treatment strategy is to eliminate gluten from the diet. Recent studies have highlighted a role for the small intestinal microbiota in modulating gluten-mediated immunopathology. *Pseudomonas aeruginosa* expresses elastase, which results in peptides that are more easily translocated across the epithelium and are more immunogenic. In addition, *Lactobacillus* spp. degrade gluten peptides produced by human and other bacteria, reducing their immunogenicity (17). AHR ligands produced through gut microbial metabolism of tryptophan reduce immunopathology in genetically susceptible, gluten-challenged mice (81). *Bifidobacterium* species like *B. lactis* and *B. longum* reduce production of inflammatory cytokines in gluten-challenged cell lines (113).

These two examples—lactose intolerance and celiac disease—highlight that, even though gut bacteria are not the primary drivers of disease in genetic conditions, they can modulate symptoms, disease course, and treatment efficacy in these conditions.

## 7. PERSPECTIVE

This review has discussed several important concepts and summarized available data on the role of the gut microbiota as a mediator of the effect of diet on host function. Diet-derived metabolites vary according to the activity of different metabolic pathways in the host and in the gut microbiota, which in turn determine the biologic effects of diet. Additional microbial metabolites can have distinct effects on multiple physiologic functions. Thus, the same metabolite can influence the pathophysiology of multiple diseases, which may explain the common finding of decreased levels of certain metabolites such as butyrate across multiple chronic diseases. It also explains why the same diet intervention may show benefits in different diseases.

**Figure 3:**
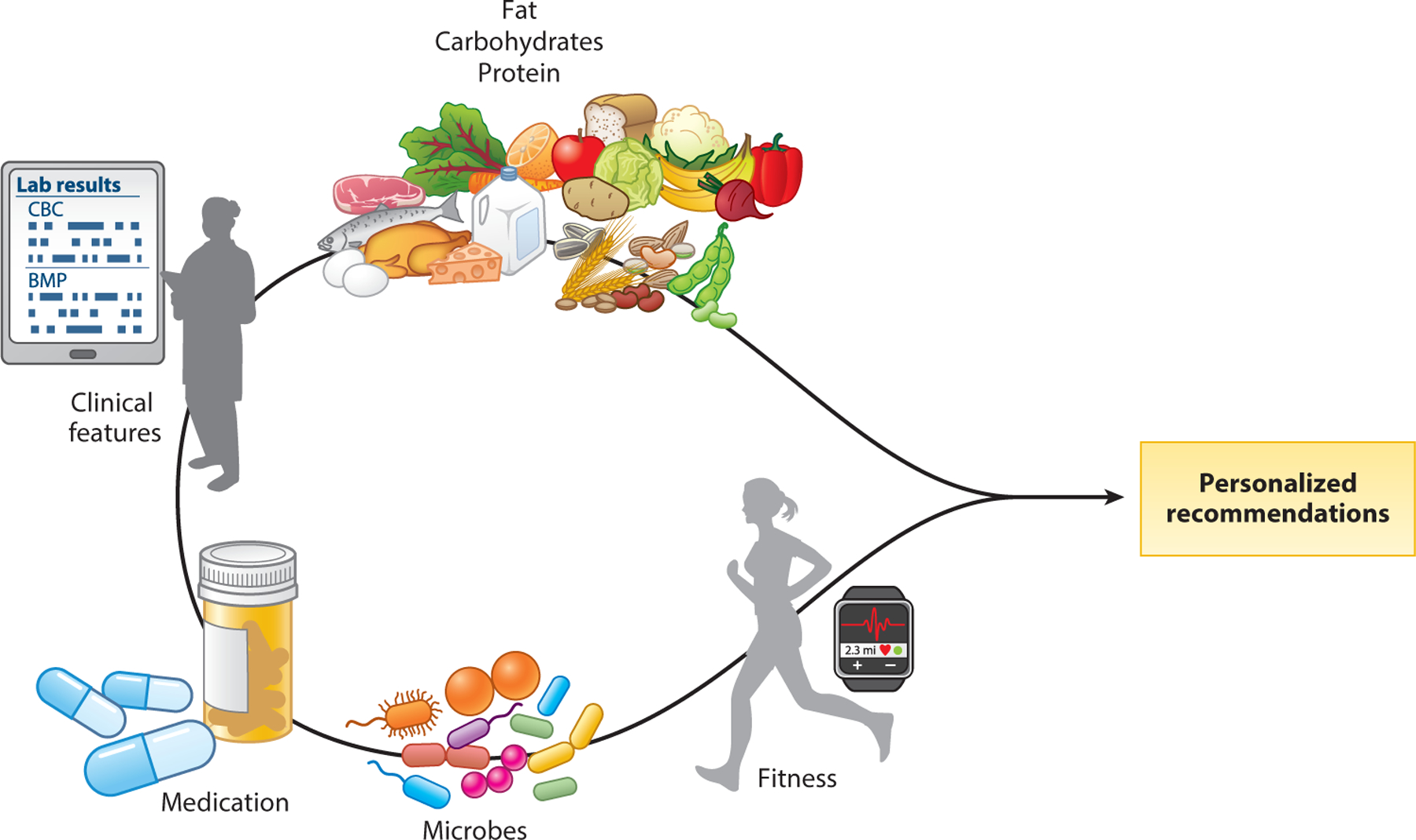
Features of the host, environment, and gut microbiota can help in developing personalized nutrition recommendations. Different types of dietary macro- and micronutrients, together with an individual’s clinical features, exercise, medications/supplements, and gut microbiota, determine the repertoire of microbial bioactive metabolites in the gut, with distinct effects on host physiology. All of these factors and as-yet-unidentified factors need to be considered in order to develop personalized nutrition recommendations.

We are still in the early stages of investigating how bioactive molecules resulting from diet–host–gut microbiota interaction affect pathophysiology and treatment responses in chronic GI diseases. An important consideration from the findings obtained to date is the significant interindividual variability observed when evaluating responses to diet interventions. This variability may arise from differences in bioavailable nutrients in the diet as a result of differences in composition and processing (e.g., cooking), genetic polymorphisms affecting metabolic pathways in the host or underlying host immune status, and differences in metabolic capabilities of the gut microbiota. In addition, other environment and host factors likely contribute to the responses. As we look to the future, we will need to consider all of these factors to be able to provide personalized dietary recommendations to our patients (Figure 3).

## Figures and Tables

**Table 1:** Human trials investigating the efficacy of MD and LFD in IBD and IBS

Disease(s)	Participant number	Dietary regimen	Primary outcome	Secondary outcome(s)	Key findings	Reference
**MD**						
CD	96	MD and SCD for 6 weeks	Symptomatic remission at week 6	FC (<250 μ/g) and CRP levels (high-sensitivity CRP < 5 mg/L)	Clinical remission: 46.5% with SCD and 43.5% with MD (*p* = 0.77) at week 6 FC response: 34.8% with SCD and 30.8% with MD (*p* = 0.83) CRP response: 5.4% with SCD and 3.6% with MD (*p* = 0.68)	Lewis et al. (87)
CD and UC	100 (MD, 50; control diet, 50)	MD with good adherence over 12 weeks with a KIDMED eight-point score	Clinical remission (PCDAI and PUCAI < 10)	CRP, calprotectin, TNF-α, IL-17, IL-12, and IL-13	PCDAI: MD group, 6.4 ± 8.1; control group, 10.8 ± 7.4 (*p* = 0.02) PUCAI: MD group, 7.6 ± 11.2; control group, 9.2 ± 7.5 (*p* = 0.04). Significantly low CRP, TNF-α, IL-17, IL-12, and IL-23 in MD group	El Amrousy et al. (41)
CD and UC	142 (UC, 84; CD, 58)	MD for 6 months	Anthropometric parameters (BMI, waist circumference, lean and fat body mass, and visceral fat), serum lipid profile, liver function and steatosis, and intestinal disease activity	None	MD improved BMI (*p* = 0.002), waist circumference (*p* = 0.041), and liver steatosis (*p* = 0.0016). Fewer UC and CD patients had active disease. Improvement in QoL in both UC and CD	Chicco et al. (26)
**LFD**						
	89 (LFD, 44; ND, 45)	LFD for 6 weeks	Response rate in IBS-SSS	QoL	Larger proportion of responders in LFD group (81%) than in ND group (46%) (*p* < 0.01) LFD group showed lower median IBS-SSS than ND group (*p* = 0.02). Improvement in QoL (greater SIBDQ in LFD group)	Pedersen et al. (114)
CD and UC	72 (CD, 52; UC, 20)	LFD for 3 months	FGS	None	Abdominal pain, bloating, wind, and diarrhea improved in CD and UC (*p* < 0.02).	Gearry et al. (50)
IBD	88	LFD	FGS by Gastrointestinal Symptoms Rating Scale	Stool output assessed by Bristol Stool Form Scale	Relief from FGS with LFD when compared with baseline (*p* < 0.001) Improvement in stool frequency (*p* = 0.001) and form (*p* = 0.002)	Prince et al. (119)
IBS	Meta-analysis of two RCTs for GFD(*n* = 111) and seven RCTs for LFD (*n* = 397)	LFD and GFD	Efficacy of exclusion diets (GFD and LFD) on global IBS symptoms	None	No significant effect of GFD on reduced IBS symptoms LFD associated with reduced IBS symptoms; however, the quality of the data was very low.	Dionne et al. (34)
IBS	Total of 99 (33 per dietary arm)	LFD, GFD, and traditional diet for 4 weeks	Response rate in IBS-SSS	Acceptability of food, stool dysbiosis	Similar improvements in IBS-SSS items regardless of their allocated diet Traditional diet was easier to follow. Stool dysbiosis indices were similar across the diets.	Rej et al. (123)

Abbreviations: BMI, body mass index; CD, Crohn’s disease; CRP, C-reactive protein; FC, fecal calprotectin; FGS, functional gastrointestinal symptoms; GFD, gluten-free diet; IBD, inflammatory bowel disease; IBS, irritable bowel syndrome; IBS-SSS, IBS Severity Scoring System; IL, interleukin; KIDMED, Mediterranean Diet Quality Index for Children and Teenagers; LFD, low-FODMAP (fermentable oligo-, di-, and monosaccharides and polyols) diet; MD, Mediterranean diet; ND, normal diet; PCDAI, Pediatric Crohn’s Disease Activity Index; PUCAI, Pediatric Ulcerative Colitis Activity Index; QoL, quality of life; RCT, randomized controlled trial; SCD, specific-carbohydrate diet; SIBDQ, Short IBD Quality of Life Questionnaire; TNF, tumor necrosis factor; UC, ulcerative colitis.

**Table 2:** Clinical trials^a^ of prebiotics and synbiotics in IBS and IBD

Participants^b^	Study protocol	Key findings	Reference
***Prebiotics***			
FGID with flatulence	Prebiotic group: 2.8 g/day Bimuno containing 1.37 g β-galacto-oligosaccharide plus a Mediterranean-type diet (*n* = 19) Placebo group: 2.8 g xylose plus a low-FODMAP diet (*n* = 21) Duration: 4 weeks Follow-up: 2 weeks	Increased *Bifidobacterium* and decreased *Bilophila wadsworthia* in patients in prebiotic group; opposite for placebo group before and after treatment Lower symptom scores for pain, distension, and bloating in both groups Symptoms recurred when the low-FODMAP diet was discontinued in the placebo group, but not in the prebiotic group after prebiotic discontinuation.	Huaman et al. (66)
All types of IBS	Prebiotic group: PHGG (*n* = 49) Placebo group: maltodextrin (*n* = 59) Dose and duration: 3 g/day for 7 days, then 6 g/day for 11 weeks Follow-up: 4 weeks	Decreased gas and bloating in IBS patients during and 4 weeks after PHGG treatment, but other symptoms and the severity score were not affected.	Niv et al. (110)
All types of IBS with rectal hypersensitivity	Prebiotic group: scFOS (*n* = 41) Placebo group: maltodextrin (*n* = 38) Dose and duration: 2.5 g, twice per day, for 4 weeks	Increased *Bifidobacterium* in scFOS group; increased *Roseburia* spp. in placebo group Rectal sensitivity was improved in both groups, and the prebiotic effect was more pronounced in IBS-C patients. IBS severity score (abdominal pain) was improved in both groups, and proportional reduction in patients feeling pain was more pronounced in the scFOS group. Anxiety and depression scores were improved by both treatments, more so in the scFOS group.	Azpiroz et al. (8)
UC	Prebiotic group: 10 g GBF, 3 times per day, with conventional medication (*n* = 23) Control group: conventional medication only (*n* = 23) Duration: 2 months	Decreased serum CRP in GBF group Decreased abdominal pain and cramping in GBF group	Faghfoori et al. (44)
CD (inactive or mild/moderate active)	Prebiotic group: OF-IN (*n* = 34) Placebo group (nonspecified) (*n* = 33) Dose and duration: 10 g, twice per day, for 4 weeks	Decreased *Ruminococcus gnavus* and increased *B. longum* in OF-IN group Positive correlation between improvement in disease activity and increase in *B. longum* abundance	Joossens et al. (71)
CD (active)	Prebiotic group: FOS (*n* = 54) Placebo group: maltodextrin (*n* = 49) Dose and duration: 7.5 g, twice per day, for 4 weeks	Lower IBD questionnaire score in the FOS group No difference in CD activity index, CRP, or fecal concentration of bifidobacteria or *F. prausnitzii* between treatment groups Increased severity of flatulence, abdominal pain, and borborygmi in FOS group Decreased IL-6^+^ lamina propria dendritic cells and IL-10 staining on dendritic cells in FOS group	Benjamin et al. (11)
IBS-D	Synbiotic group: mixture of *B. lactis*, *B. longum*, *B. bifidum*, *L. acidophilus*, and *L. rhamnosus*, with 947 mg scFOS (*n* = 35) Placebo group: 978 mg maltodextrin (*n* = 33) Dose and duration: 5.00 × 10^9^ CFU, twice per day, for 4 + 4 weeks (primary and secondary endpoints)	Improved global symptoms and severity, as well as flatulence and bowel habit, in synbiotic group at both endpoints	Skrzydło-Radomańska et al. (134)
IBS-C	Synbiotic group: 350 mL of sterilized probiotic with *L. helveticus* and 5.85 g polydextrose (*n* = 79) Control group: 350 mL of sterilized probiotic with *L. helveticus* (*n* = 84) Duration: 7 days	Faster gut transit and lower fecal pH in both groups Higher fecal weight in synbiotic group but lower in control group Constipation-related symptoms were improved in both groups.	Bahrudin et al. (9)
IBS-C	Synbiotic group: *L. acidophilus* La-5 (1.8 × 10^7^ CFU/g), *B. animalis* subsp. *lactis* BB-12 (2.5 × 10^7^ CFU/g), *S. thermophilus*, and 90% inulin plus 10% oligofructose (*n* = 11) Placebo group: heat-treated fermented milk (*n* = 19) Duration: 4 weeks Follow-up: 1 week	Transient increase in abundance of used probiotic strains in synbiotic group No functional study	Bogovič Matijašić et al. (14)
All types of IBS	Synbiotic group: Lactol^®^ (including *Bacillus coagulans* at 15 × 10^7^ spores and FOS at 100 mg) (*n* = 23) Placebo group: lactose starch and tartrazine (*n* = 33) Dose and duration: 3 times/day for 12 weeks Follow-up: 9 months	Decreased frequency of abdominal pain, diarrhea, and constipation in synbiotic group (diarrhea is specific to synbiotic group, improvement of constipation did not differ between groups) Decreased abdominal pain and increased constipation frequency in synbiotic group during follow-up, while both abdominal pain and diarrhea increased in placebo group	Rogha et al. (125)
All types of IBS	Synbiotic group: Probinul (lyophilized bacteria including *L*. *plantarum* and *S. thermophilus* of 5 × 10^9^ CFU each; *L. casei* subsp. *rhamnosus* and *L. gasseri* at 2 × 10^9^ CFU each; *B. infantis*, *B. longum*, *L. acidophilus*, *L. salivarus*, and *L. sporogenes* at 1 × 10^9^ CFU each) with 2.2g inulin and 1.3 g tapioca-resistant starch (*n* = 32) Placebo group (nonspecified) (*n* = 32) Dose and duration: 5 g, twice per day, for 4 weeks	Decreased flatulence and increased transit time with higher QoL in synbiotic group	Cappello et al. (18)
All types of IBS	Synbiotic group: yogurt containing ≥10^11^ CFU/150 mL *B. animalis* subsp. *lactis* Bb-12 with *Bifidobacterium* enhancer, acacia dietary fiber, ≥3 × 10^9^ CFU/150 mL *S. thermophilus*, and ≥10^9^ CFU/150 mL *L. acidophilus* (*n* = 58) Control group: traditional yogurt containing ≥10^10^ CFU/150 mL *B. animalis* subsp. *lactis* Bb-12, with ≥3 × 10^9^ CFU/150 mL *S. thermophilus* and ≥10^9^ CFU/150 mL *L. acidophilus*; no extrafunctional ingredients (*n* = 59) Dose and duration: twice per day for 8 weeks	Overall higher improvement in IBS symptoms and bowel habit satisfaction in synbiotic versus control group, with better alleviation of symptoms in IBS-C patients on synbiotic yogurt and more satisfaction of bowel habit in IBS-D patients on synbiotic yogurt compared with those on control yogurt	Min et al. (98)
UC (mild to moderate active)	Synbiotic group: 3 × 10^9^ CFU of *E. faecium*, *L. plantarum*, *S. thermophilus*, *B. lactis*, *L. acidophilus*, and *B. longum*, with 225 mg/dose FOS (*n* = 18) Placebo group (nonspecified) (*n* = 18) Dose and duration: twice per day for 8 weeks	Lower serum CRP in synbiotic group Decreased symptom severity but greater remission in synbiotic group	Altun et al. (3)

a Includes only studies published between 2010 and 2022, with randomized, double-blind, and placebo-controlled designs.

b All IBS patients were selected on the basis of Rome III criteria.

Abbreviations: CD, Crohn’s disease; CFU, colony-forming units; CRP, C-reactive protein; FGID, functional gastrointestinal disorders; FODMAP, fermentable oligo-, di-, and monosaccharides and polyols; FOS, fructo-oligosaccharides; GBF, germinated barley foodstuff; IBS-C, constipation-predominant irritable bowel syndrome; IBS-D, diarrhea-predominant irritable bowel syndrome; IL, interleukin; OF-IN, oligofructose-enriched inulin; PHGG, partially hydrolyzed guar gum; QoL, quality of life; scFOS, short-chain fructo-oligosaccharides; UC, ulcerative colitis.
